# Epstein - Barr Virus Transforming Protein LMP-1 Alters B Cells Gene Expression by Promoting Accumulation of the Oncoprotein ΔNp73α

**DOI:** 10.1371/journal.ppat.1003186

**Published:** 2013-03-14

**Authors:** Rosita Accardi, Ikbal Fathallah, Henri Gruffat, Giuseppe Mariggiò, Florence Le Calvez-Kelm, Catherine Voegele, Birke Bartosch, Hector Hernandez-Vargas, James McKay, Bakary S. Sylla, Evelyne Manet, Massimo Tommasino

**Affiliations:** 1 International Agency for Research on Cancer, World Health Organization, Lyon, France; 2 INSERM U758, Lyon, France; 3 Ecole Normale Supérieure de Lyon, Lyon, France; 4 Université Claude Bernard Lyon I, Lyon, France; 5 CRCL, INSERM U1052, CNRS 5286, Université de Lyon, Lyon, France; University of Wisconsin-Madison, United States of America

## Abstract

Many studies have proved that oncogenic viruses develop redundant mechanisms to alter the functions of the tumor suppressor p53. Here we show that Epstein-Barr virus (EBV), via the oncoprotein LMP-1, induces the expression of ΔNp73α, a strong antagonist of p53. This phenomenon is mediated by the LMP-1 dependent activation of c-Jun NH2-terminal kinase 1 (JNK-1) which in turn favours the recruitment of p73 to ΔNp73α promoter. A specific chemical inhibitor of JNK-1 or silencing JNK-1 expression strongly down-regulated ΔNp73α mRNA levels in LMP-1-containing cells. Accordingly, LMP-1 mutants deficient to activate JNK-1 did not induce ΔNp73α accumulation. The recruitment of p73 to the ΔNp73α promoter correlated with the displacement of the histone-lysine N-methyltransferase EZH2 which is part of the transcriptional repressive polycomb 2 complex. Inhibition of ΔNp73α expression in lymphoblastoid cells (LCLs) led to the stimulation of apoptosis and up-regulation of a large number of cellular genes as determined by whole transcriptome shotgun sequencing (RNA-seq). In particular, the expression of genes encoding products known to play anti-proliferative/pro-apoptotic functions, as well as genes known to be deregulated in different B cells malignancy, was altered by ΔNp73α down-regulation. Together, these findings reveal a novel EBV mechanism that appears to play an important role in the transformation of primary B cells.

## Author Summary

Approximately 20% of worldwide human cancers have been associated with viral infections. Many oncogenic viruses exert their transforming properties by inactivating the products of tumour suppressor genes. One of the best characterized events induced by ongocenic viruses is the inactivation of the transcriptional factors p53. The mucosal high-risk HPV types, EBV, HTLV-1 and KSHV, via their viral proteins, are able to target p53 by distinct mechanisms. We have recently described a novel p53 inactivation mechanism of some cutaneous beta HPV types which have been suggested to be associated with skin carcinogenesis. Beta HPV38 induces accumulation of the p53 antagonist, ΔNp73α which in turn silences the expression of the p53-regulated genes. Here we report that also EBV, via the oncoprotein LMP-1, induces the expression of ΔNp73α which is dependent on the recruitment of p73 on ΔNp73 promoter and the activation of JNK-1. The recruitment of p73 to the ΔNp73 promoter correlated with the displacement of the histone-lysine N-methyltransferase EZH2 which is part of a transcriptional repressive polycomb 2 complex. We also show that ΔNp73α plays an important role in transformation of primary human B cells and regulates the expression of a large number of cellular genes that encode proteins linked to cancer development, including lymphomagenesis.

## Introduction

Epstein-Barr virus, also known as human herpesvirus 4 (HHV4), belongs to the gammaherpesvirus family and is largely spread as it can be detected in 90% of the worldwide population. EBV infects B cells and, in most cases, does not lead to any clinical manifestations. However, when EBV infection occurs during adolescence or young adulthood, it may cause infectious mononucleosis, a benign lymphoproliferative disease. A minority of EBV infections result in the development of several types of human B cell malignancies, including Burkitt's lymphoma (BL), Hodgkin and non-Hodgkin lymphomas [1]. In addition, EBV has been clearly associated with epithelial cancers, i.e. nasopharyngeal carcinoma (NPC) and a sub-set of gastric carcinoma [1]. The risk of developing EBV-induced malignancies is significantly increased in immuno-compromised individuals, such as AIDS patients and organ-transplant recipients. *In vitro* EBV efficiently infects human resting B cells and transforms them into proliferating lymphoblastoid cell lines (LCLs) [2]. Similar to other herpesviruses, the EBV life cycle includes a latent and non-productive phase, as well as a lytic phase leading to the production of the virus progeny. After primary infection, EBV persists lifelong in a latent state in a sub-population of resting memory B cells [3]. Recent studies led to a model of EBV persistence whereby different viral transcription programs were used within the context of the normal biology of B lymphocytes in order to carry out its life cycle [4], [5]. Eleven genes can be expressed in the latency phases, namely the EBV nuclear antigens (EBNA) 1, 2, 3A, 3B, 3C, LP, the latent membrane proteins (LMP) 1, 2A, 2B, the untranslated EBER-1 and EBER-2 RNAs, as well as multiple microRNAs [2]. Based on the expression pattern of the different latency genes, four latency phases have been identified so far. Type I latency is normally present in Burkitt's lymphoma and is associated with the expression of EBNA-1 as well as EBERs and miRNAs. Type II latency is frequently detected in Hodgkin's lymphoma and nasopharyngeal carcinoma, and is linked to the expression of EBNA-1, LMP-1, LMP-2A, LMP-2B, EBERs and miRNAs. Type III latency is characterized by the expression of all 11 latency genes and is mainly found in lymphoproliferative diseases in immunocompromised individuals and in *in vitro* EBV-transformed LCLs. Finally, type IV latency is associated with the infectious mononucleosis and is less well defined, since the expression pattern of the latency genes may differ in different patients [6].

LMP-1 is the major EBV oncoprotein and displays transforming activities in *in vitro* and *in vivo* models [2]. It is an integral membrane protein composed of a short cytoplasmic amino-terminal domain, six hydrophobic transmembrane domains, and a cytoplasmic carboxy-terminal domain [2]. LMP-1 exerts its transforming properties by functioning as a member of the tumour necrosis factor receptor (TNFR) superfamily leading to the a constitutive activation of several cellular signaling pathways [7]–[9]. In particular, LMP-1 activates the nuclear factor-kappa B (NF-κB) signaling pathway, thus promoting cell growth and inhibition of apoptosis. In addition to LMP-1, other EBV latent proteins, i.e. EBNA-2, EBNA-3A and EBNA-3C, are involved in the immortalization of primary B cells. The role of EBNA-2 is mainly mediated by its ability to modulate the transcription of host and viral genes, while EBNA-3C plays a direct role in cellular transformation by inactivating the products of tumor suppressor genes, such as retinoblastoma (pRb) and p53 [10]–[14]. Interestingly, as for other oncogenic viruses, e.g. human papillomavirus type 16 (HPV16) [15], EBV developed multiple and redundant mechanisms to inactivate p53-regulated pathways. Indeed, EBNA-3C is able to alter the p53 transcription activity via direct binding as well as by inducing stabilization of p53 inhibitors, such as mdm2 and Gemin3 [10], [12], [14].

p73 is a closely p53-related transcription factor that shows functional similarity to p53 [16], [17]. The impact of EBV on p73 has been poorly investigated so far, although initial findings indicate that, similarly to p53, p73 is targeted by EBV. Indeed, p73 expression was found down-regulated in EBV-positive gastric carcinoma by heavy methylation of CpG islands within its promoter [18]. In addition, it has been recently shown that EBNA-3C attenuates p73 expression in LCLs by targeting the transcription factor E2F-1 [19]. However, it is likely that, in line with the previous findings on p53, EBV has developed multiple mechanisms to alter p73 function. In addition, due to the variability in the expression pattern of the latent genes in EBV-positive cancer cells, it is possible that more than one viral protein has the ability to target the p53/p73 pathway.

We have previously shown that cutaneous HPV38 which belongs to an HPV subgroup potentially associated with the development of non-melanoma skin cancer (NMSC), is able to induce the accumulation of ΔNp73α which in turn alters the transcriptional functions of p53 and p73 [20], [21]. ΔNp73α is a p73 isoform that lacks transactivation (TA) domain and is accumulated in several tumors [22]. Most importantly, increase in ΔNp73α protein levels correlates with poor outcome of the disease and bad response to therapy [23]–[25].

Here we show that EBV LMP-1 activates ΔNp73α expression in B cells by favoring the recruitment of p73 to ΔNp73 promoter. This phenomenon appeared to be mediated by c-Jun NH2-terminal kinase 1 (JNK-1), and resulted in the inhibition of p53-regulated genes encoding key anti-proliferative regulators.

## Results

### EBV induces ΔNp73 transcriptional activation in B cells

**Figure 1:**
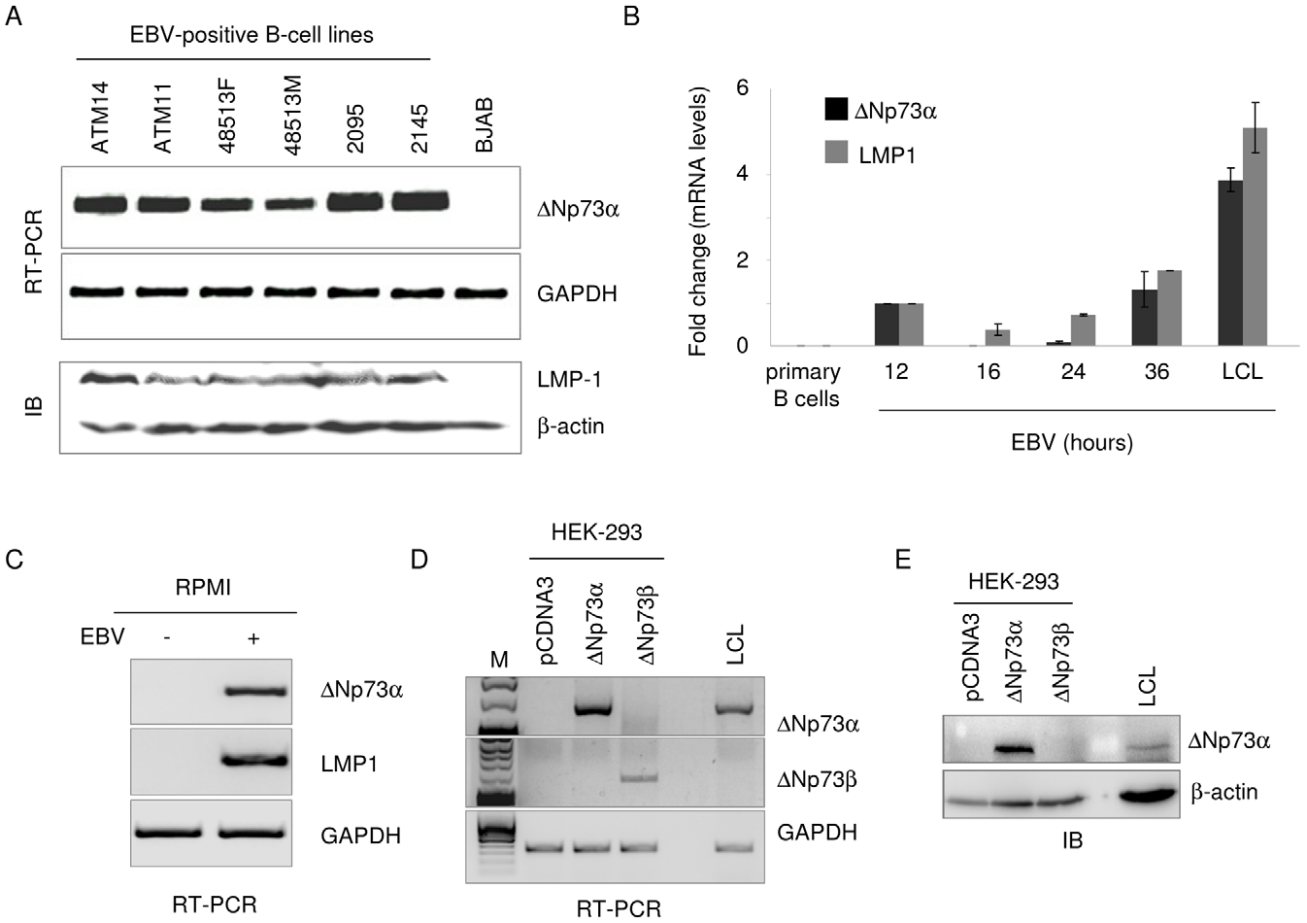
EBV induces ΔNp73 transcriptional activation in B cells. (**A**) EBV positive and negative immortalized B cells were collected and processed for total RNA and protein extraction. The levels of ΔNp73 and GAPDH were determined by RT-PCR (upper panels). Protein extracts were analyzed by immunoblotting with indicated antibodies (lower panel). (**B**) Primary B cells were infected with EBV-GFP recombinant virus. Infected cells were collected at the indicated time points and processed for RNA extraction. The levels of ΔNp73, LMP-1 and GAPDH were determined by quatitative RT-PCR. The data are the mean of three independent experiments. (**C**) RPMI cells were infected with EBV and 2 weeks after infection were collected and processed for RNA extraction and RT-PCR analysis. (**D**) Total RNA was prepared from LCL and HEK 293 cells transduced with empty retrovirus or expressing ΔNp73α or ΔNp73β isoform RT-PCR was performed using specific ΔNp73 isoform primers. M indicates the DNA marker used to confirm the size of the PCR fragments (MassRuler DNA Ladder Mix, Fermentas). (**E**) Whole cell extracts were prepared from LCL and HEK293 cells, the latter were transfected with pcDNA empty or with pcDNA- ΔNp73α and ΔNp73β. 40 µg of cell lysates were analyzed by immunoblotting with p73 or β-actin antibody.

Several isoforms of ΔNp73 have been identified, that can be generated by alternative splicing at the 5′ region of the p73 mRNA or by transcriptional initiation from a promoter (p2) within the p73 gene [26], [27]. We have previously shown that HPV38 induces the accumulation of ΔNp73α mRNA generated by the p2 promoter [20]. We therefore evaluated whether the levels of this specific ΔNp73 transcript were induced by EBV. ΔNp73α mRNA levels were determined in EBV-positive and EBV-negative B-cell lines by RT-PCR using specific primers. Six LCLs expressed high levels of ΔNp73α transcript, while no signal was detected in the EBV-negative B lymphoma cell line BJAB (Figure 1A). To assess that the over-expression of ΔNp73α was indeed linked to EBV infection, we infected primary B cells with recombinant EBV and analyzed ΔNp73α mRNA levels by quantitative RT-PCR. As previously shown, ΔNp73α is not expressed in primary B cells (Figure 1B) [28]. In contrast, an increase in ΔNp73 mRNA levels was observed between 12–36 hours post-EBV infection which correlated with LMP1 transcript levels (Figure 1B). ΔNp73 mRNA accumulation was also observed in cancer B-cell lines, RPMI, upon EBV infection cells (Figure 1C). Several C-terminus ΔNp73 isoforms have been characterized, that are generated by alternative splicing (α, β, γ, ε). The isoform alpha plays a key role in altering the p53/p73 functions and is over-expressed in several human cancers [29]. RT-PCR experiments with specific ΔNp73 isoform primers confirmed that alpha, and not beta, ΔNp73 is expressed in EBV-infected B-cells (Figure 1D). Accordingly, immunoblotting with a p73 antibody revealed a 65–70 kD protein band in LCLs that co-migrated with the ΔNp73α ectopically expressed in HEK 293 cells (Figure 1E).

Taken together, these data showed that EBV specifically activates ΔNp73α transcription in primary and cancer B-cells.

### LMP-1 plays a central role in EBV-mediated up-regulation of ΔNp73α

**Figure 2:**
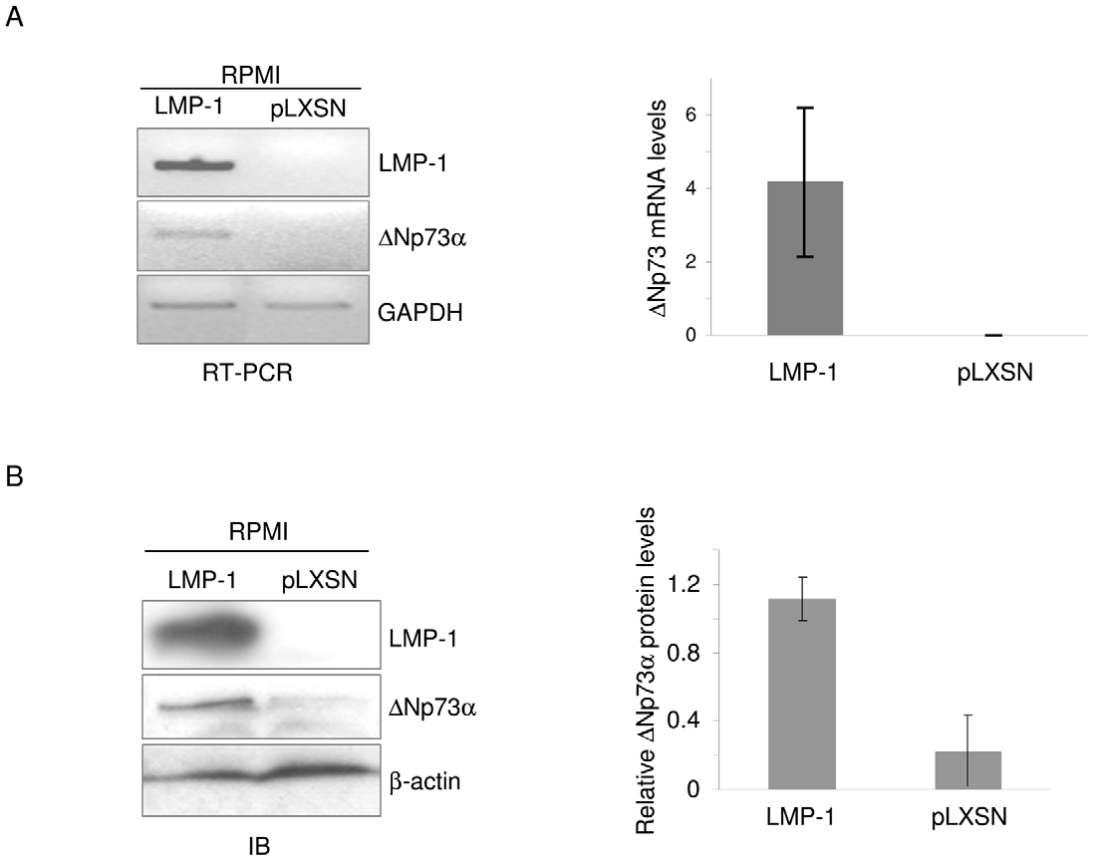
EBV LMP-1 induces up-regulation of ΔNp73α. (**A and B**) RPMI-pLXSN (pLXSN) and RPMI-pLXSN-LMP-1 (LMP-1) cells were collected and processed for the preparation of total RNA or protein extract. RNA was retro-trancribed to cDNA and ΔNp73, LMP-1 and GAPDH mRNA levels were measured by RT-PCR (A right panel) or quantitative PCR (A left panel). Protein extracts were analyzed by immunoblotting using the indicated antibodies (B left panel). The ΔNp73α protein signal was quantified by Quantity one (Biorad), normalized on the levels of β-actin, and the values obtained were reported in the histogram (B left panel). The data are the mean of three independent experiments.

**Figure 3:**
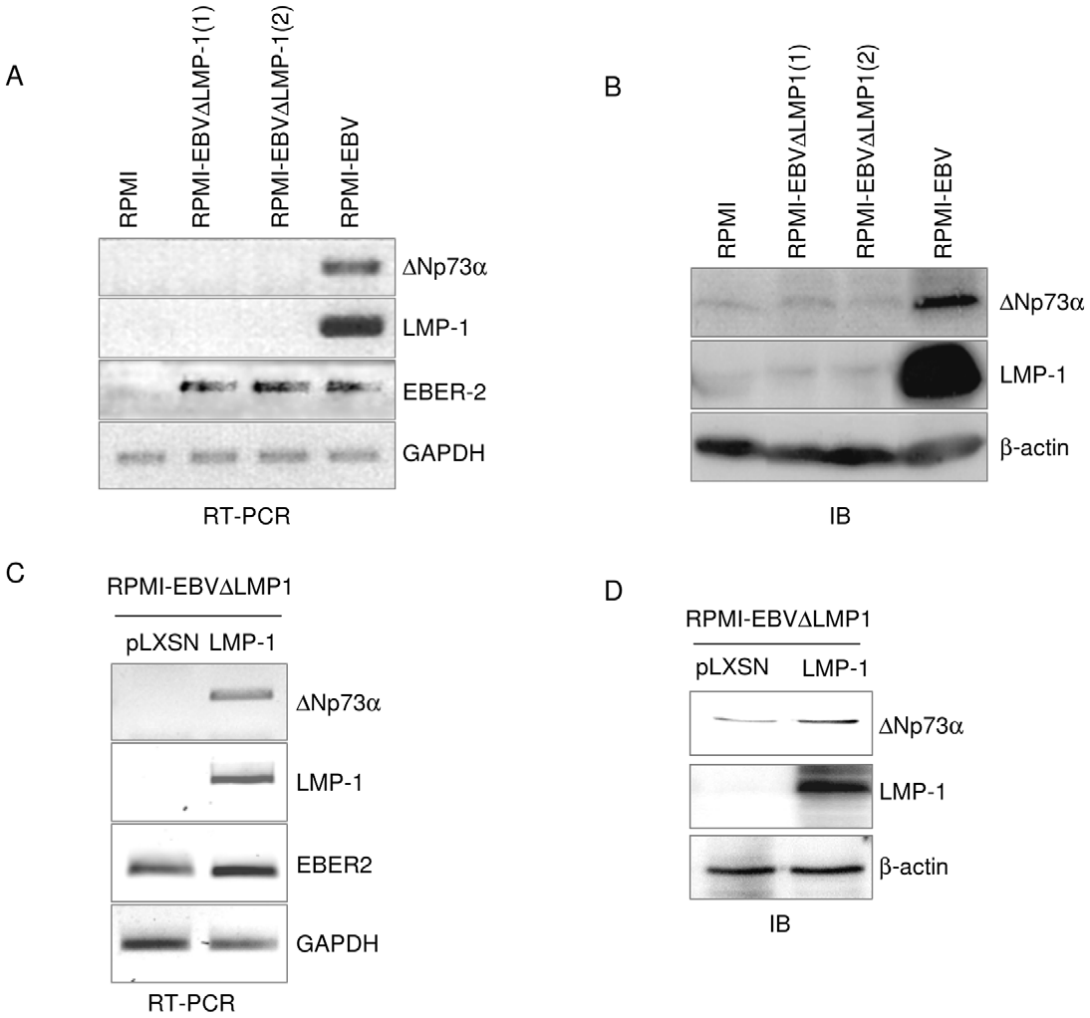
Expression of LMP-1 in cells infected by EBVΔLMP-1 restores ΔNp73α levels. (**A and B**) Two independent infections of RPMI with EBVΔLMP-1 recombinant virus, not infected RPMI and RPMI carrying the wild-type EBV genome were collected and processed to obtain total RNA or whole cell extracts. (A) The levels of ΔNp73, LMP-1, EBER-2 and GAPDH transcripts were determined by RT-PCR. (B) Protein extracts were analyzed by immunoblotting using the indicated antibodies. Please note that lane 1 to 3 and lane 4 have been joined from 2 different areas of the same immunoblot. (**C and D**) Total RNA and total cell extracts from RPMI- EBVΔLMP-1 were transduced with pLXSN or pLXSN-LMP-1 retroviruses were prepared. (C) Transcript levels of ΔNp73α, LMP-1, EBER-2 and GAPDH were measured by RT-PCR. (D) Protein extracts were analyzed by immunoblotting for the levels of ΔNp73, LMP-1. β-actin was used as loading control.

Studies on other oncogenic viruses demonstrated that alterations of p53-regulated pathways are normally induced by the viral oncoproteins [15], [30]. Figure 1B showed a correlation between ΔNp73α and LMP-1 expression levels, supporting the possible involvement of the viral oncoprotein in ΔNp73α up-regulation. Therefore, we determined later whether the major EBV transforming protein, LMP-1, was responsible for ΔNp73α accumulation. RPMI cells were transduced with empty (pLXSN) or LMP-1 expressing retrovirus (pLXSN-LMP-1) and ΔNp73α transcript and protein levels were determined by RT-PCR and immunoblotting, respectively. Both ΔNp73α mRNA and protein levels were strongly increased in RPMI/LMP-1 cells in comparison to cells infected with the empty retroviral vector (Figures 2A and B). To further demonstrate the role of LMP-1 in ΔNp73α up-regulation, we infected RPMI cells with a wild-type or mutated EBV, in which the LMP-1 gene was deleted (EBVΔLMP-1). Two RPMI/EBVΔLMP-1 cell lines were generated by two independent infections and ΔNp73α expression levels were compared with the ones of mock infected cells as well as RPMI/EBV. Deletion of LMP-1 gene from the EBV genome abolished ΔNp73α up-regulation, as shown by RT-PCR and immunoblot analyses (Figures 3 A and B). Transduction of RPMI/EBVΔLMP-1 cells with a recombinant retrovirus expressing LMP-1 (pLXSN LMP-1) restored the ability of EBV to promote ΔNp73α mRNA and protein accumulation (Figures 3C and D).

In summary, these data highlight the central role of LMP-1 in EBV-mediated ΔNp73α up-regulation.

### p73 activates ΔNp73α expression in LMP-1-expressing cells

**Figure 4:**
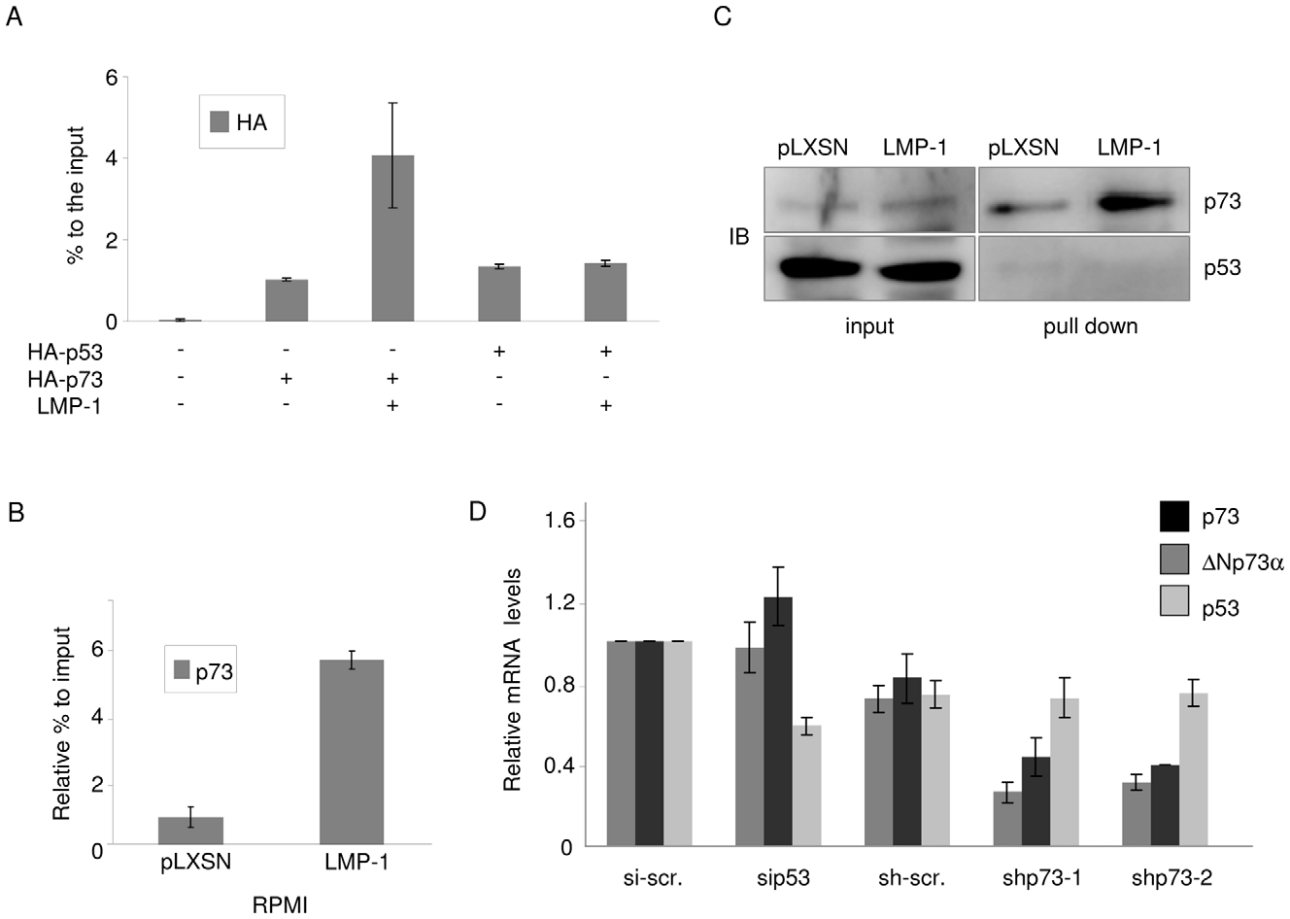
p73 is recruited to ΔNp73 in promoter LMP-1 expressing cells. (**A**) SaOS-2 cells were transfected with different pcDNA3 constructs in the indicated combinations. After 36 hours, ChIP was performed using an anti HA-tag antibody and followed by real-time PCR, using primers flanking the p53-RE within the ΔNp73 promoter. Simultaneously, 1/10 of the total chromatin was processed. The values in the histogram were obtained by dividing for each sample the amount of ΔNp73 promoter, which is bound by p73-HA and p53-HA for the total amount of ΔNp73 promoter present in the input. The reported values are the mean of three independent experiments. (**B and C**) RPMI-pLXSN (pLXSN) and RPMI-pLXSN-LMP-1 (LMP-1) were fixed and processed for ChIP, or collected to perform DNA-pull down. (B) ChIP was carried out using an anti p73 antibody. The real time PCR and the quantification of the % of p73 binding to the p73-RE within the ΔNp73 promoter, was performed as explained in the legend of Figure 4A. The data are the mean of three independent experiments. The difference in the p73 levels recruited to ΔNp73α promoter in presence and absence of LMP-1 is statistically significant ((p = 0.000254) (C) After the DNA pull-down assay the levels of p53 and p73 proteins binding to biotinylated DNA probe were analysed by immunoblotting. (**D**) LCLs were transfected with scrambled RNA and siRNA for p53 (sip53) or transduced with lentivirus carrying two different OmicsLink p73 shRNAs (shp73-1, shp73-2) and scramble shRNA. Thirty-six hours after transfection or one-week post lentivirus transduction cells were collected and processed for RNA extraction and cDNA synthesis. The levels of p53, p73 and ΔNp73α transcript were determined by quantitative RT-PCR. The data are the mean of three independent experiments. The difference in mRNA ΔNp73α levels in scramble or sip53 transfected cells was not significant, while it was statistically significant between scramble and shp73-1 (p value = 0.0022) and scramble and shp73-2 (p value = 0.005).

The p73 p2 internal promoter contains a p53 responsive element (RE) which can be activated by both p53 and p73 [31]–[33]. Therefore, we evaluated whether p53 and/or p73 are involved in the regulation of p2 promoter in the presence or absence of LMP-1. Chromatin immune precipitation (ChIP) experiments using the p53 null SaOS-2 cells as experimental model showed that LMP-1 over-expression increased p73 binding affinity for the RE within the p2 promoter, while it did not influence p53 recruitment to the same site (Figure 4A). ChIP experiments with anti p73 antibody in RPMI cells transduced with empty (pLXSN) or pLXSN-LMP-1 retrovirus also showed an increased binding of p73 to the p2 promoter in presence of LMP1 (Figure 4B). In addition, DNA pull-down experiments, in which a biotinylated DNA probe containing a region of the p2 promoter encompassing the p53RE was incubated with cellular extracts of RPMI or RPMI LMP-1 cells, showed that LMP-1 increased p73 efficiency in binding DNA (Figure 4C). Silencing of p73 expression in LCL by shRNA correlated with down-regulation of ΔNp73α mRNA levels (Figure 4D). In contrast, targeting p53 with a siRNA in the same cells did not alter ΔNp73α levels (Figure 4D), indicating that p53 is not involved in the transcriptional regulation of ΔNp73α in LCLs.

We conclude from this set of data that LMP-1-mediated ΔNp73α transcriptional activation is partly due to the recruitment of p73 to the p2 promoter.

### JNK-1 is involved in LMP-1-mediated activation of ΔNp73α expression

**Figure 5:**
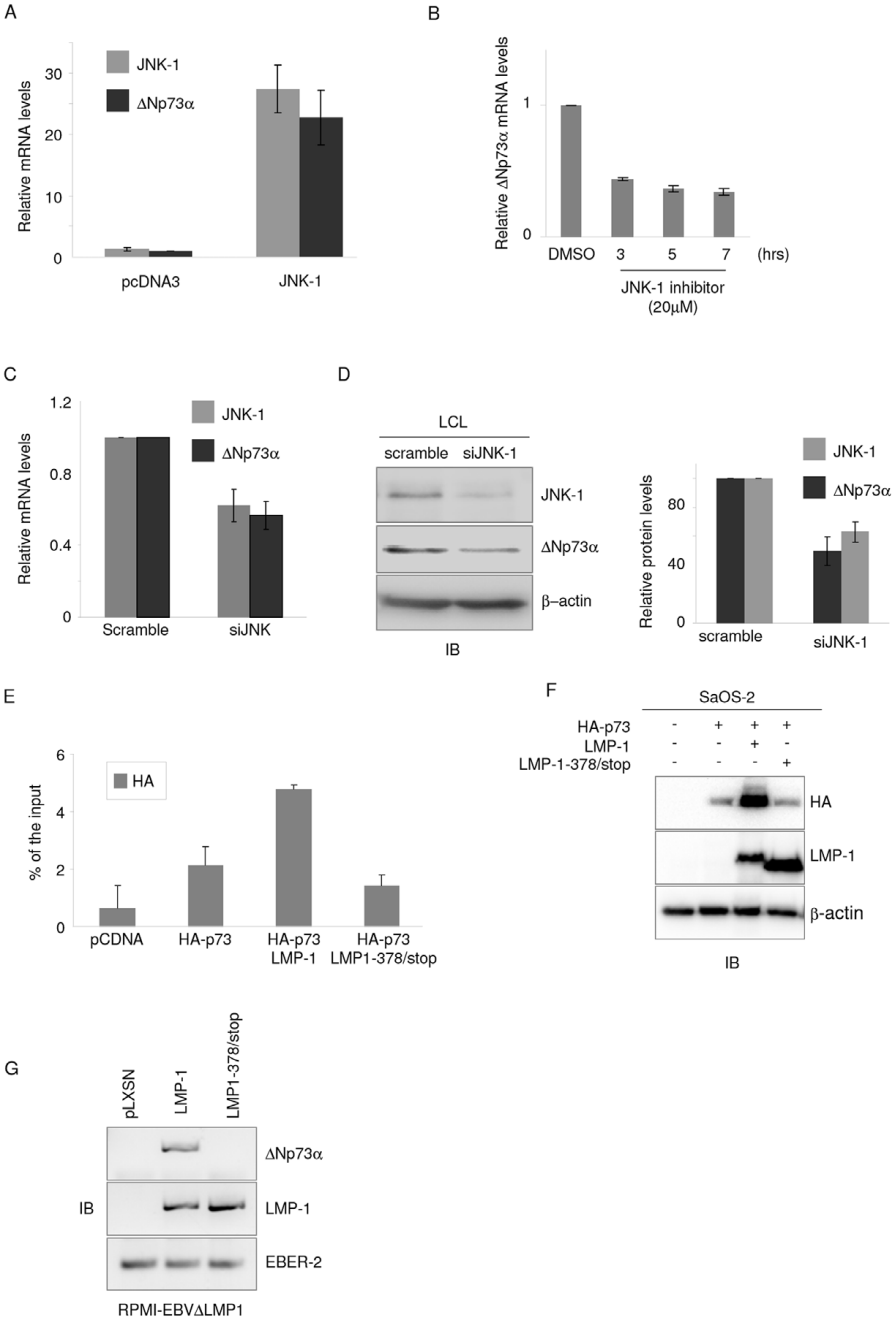
JNK-1 is involved in LMP-1-mediated activation of ΔNp73α expression. (**A**) BJAB cells were transfected with JNK-1 expression vector or empty pcDNA3 construct as control. After 36 hours cells were collected and processed for RNA extraction. The levels of ΔNp73, JNK-1 and GAPDH transcripts were determined by quantitative RT-PCR. The data are the mean of three independent experiments. The difference of ΔNp73α mRNA levels in mock cells (BJAB) and BJAB expressing JNK-1 is statistically significant (p value = 0.022). (**B**) LCLs were treated with JNK-1 inhibitor (SP600125) at 20 µM. After the indicated times cells were collected and processed for RNA extraction. ΔNp73 mRNA levels were determined by quantitative RT-PCR. The data are the mean of three independent experiments. The difference of ΔNp73α mRNA levels in LCLs cultured in presence (7 hours) or absence (DMSO) of JNK-1 inhibitor is statistically significant ((p value = 0.015). (**C and D**) Scrambled siRNA and siRNA for JNK-1 (siJNK-1) was transfected in LCLs. Thirty-six hours after transfection, cells were collected and processed for RNA or total cells proteins extraction. (C) ΔNp73 and JNK-1 transcript levels were determined by quantitative RT-PCR. The data are the mean of three independent experiments. The difference of JNK-1 or ΔNp73α mRNA levels in LCLs transfected with scramble siRNA or siJNK-1 is statistically significant (p value = 0.008 and p value = 0.0002, respectively). (D) Forty µg of whole cell lysate was analyzed by immunoblotting for the levels of ΔNp73α, JNK-1 or β-actin proteins (left panel). ΔNp73α and JNK-1 protein signal was quantified by Quantity one (Biorad), normalized on the levels of β-actin, and the values obtained were reported in the histogram (right panel). The data are the mean of three independent experiments. The difference of ΔNp73α mRNA levels in LCLs transfected with scramble siRNA or siJNK-1 is statistically significant (p value = 0.01). (**E and F**) SaOS-2 cells were transfected with different pcDNA3 constructs in the indicated combinations. After 36 hours, cells were collected and fixed for ChIP experiments performed with a HA antibody and followed by quatitative PCR (E) or analysed by immunoblot for the indicated proteins (F). (**G**) RPMI- EBVΔLMP-1 cells were transduced with pLXSN, pLXSN-LMP-1 wild-type or 378/Stop mutant retroviruses. Cells were selected for neomycin resistance, and then collected for RNA extraction. The levels of ΔNp73α, LMP-1 and EBER2 transcript were determined by RT-PCR.

It is known that LMP-1 stimulates JNK-1, which in turn leads to p73 phosphorylation and increase in its transcriptional activity [34]–[36]. We therefore determined whether JNK-1 is involved in ΔNp73α accumulation mediated by LMP-1. Ectopic levels of JNK-1 in BJAB cells induced ΔNp73α accumulation (Figure 5A). In addition, treating LCLs with a specific inhibitor of JNK (SP600125) led to a time-dependent decrease in ΔNp73α mRNA levels (Figure 5B). JNK-1 down-regulation in LCL by siRNA also led to a decrease in ΔNp73α mRNA and protein levels (Figures 5C and D).

LMP-1 transforming activities lie mostly on two distinct domains in its cytoplasmic C-terminus, namely C-terminal activation region 1 (CTAR1) (amino acids 187–231) and CTAR2 (amino acids 351–386). As CTAR2 LMP-1 mutant (LMP-1/378 stop) is unable to activate JNK-1 [37], we determined whether deletion of CTAR2 affected LMP-1 ability to promote p73 and ΔNp73α accumulation. We first transfected SaOS-2 cells with HA tagged p73 in the presence of wild-type or 378 stop-mutant LMP-1. ChIP experiments performed with an HA antibody showed that only the wild-type LMP-1 increased p73 recruitment to p2 promoter (Figure 5E). According to previous data [35], immunoblotting showed that wild-type LMP-1, but not the LMP-1 378 stop-mutant that is unable to activate JNK-1, induced p73 accumulation (Figure 5F). Finally, ΔNp73α expression was only detected in RPMI/EBVΔLMP-1 cells containing the wild-type LMP-1 and not the LMP-1 378 stop-mutant (Figure 5G).

LMP-1 is also able, via the CTAR1 and CTAR2, to activate the cellular kinase p38 which in turn activates p73 [38]–[40]. However, p38 inhibition in LCLs by a chemical inhibitor did not result in a decrease of mRNA levels of ΔNp73α (data not shown), indicating that a different mechanism is involved in the event.

Together, these data highlight a crucial role of JNK-1 in LMP-1-mediated accumulation of p73 and ΔNp73α.

### LMP-1 induces epigenetic changes on the p2 promoter

**Figure 6:**
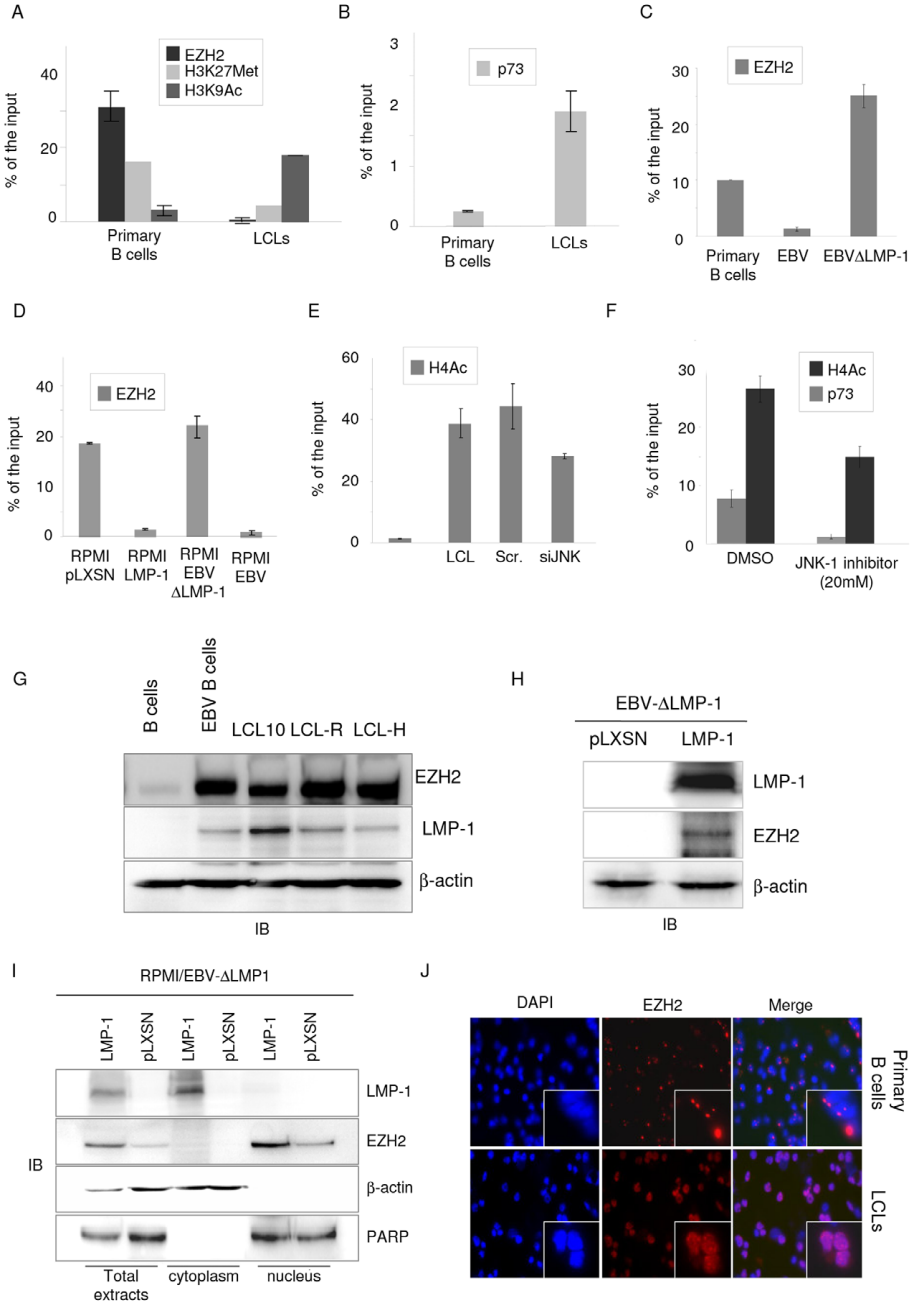
EBV infection of B cells determines epigenetic changes on the p2 promoter. (**A and B**). Primary B cells were infected and immortalized with EBV (LCL). At the day of the extraction and 2-weeks post infection, primary and immortalized B cell respectively, were fixed and processed for quantitative ChIP analysis. (A) ChIP was carried out using EZH2, Histone 3 Trimethylated Lysine 27 (H3K27), Histone 3 acetylated Lysine 9 (H3K9Ac) antibodies and IgG antibody as negative control. After ChIP the eluted DNA was used as template for quantitative PCR with primers spanning the p73RE within the ΔNp73 promoter. Part of the total chromatin fraction (1/10) was processed at the same time and used as input. After subtracting the background of the unspecific binding (ChIP for IgG), the amount of promoter specifically bound by each protein was expressed as a percentage of the total amount of ΔNp73 promoter (% of input). (B) Quantitative-ChIP for p73 was carried out and analyzed as described in the legend of Figure 6A. The data shown in (A) and (B) are the mean of three independent experiments with primary B cells of three different donors. The differences between the % of binding to the promoter in primary B cells and in LCL are significant (p value = 0.01 for EZH2, p value = 0.01 for H3K27Met, p value = 0.002 for H3K9Ac). (**C**) Primary B cells or primary B cells infected with wild-type EBV (EBV) or EBV lacking the entire LMP-1 gene (EBVΔLMP-1) were fixed and processed for EZH2 ChIP by using the LowCell ChIP Kit (Diogenode). Quantitative ChIP analysis was performed according to the manufacturers' protocol. The data are the mean of two independent experiments. The difference between the % of EZH2 binding to p2 in primary and LCL is significant (p value = 0.01). (**D**) RPMI-pLXSN (pLXSN) and RPMI-pLXSN-LMP-1 (LMP-1), RPMI EBV and RPMI EBVΔLMP-1 were fixed and processed for ChIP for EZH2 as described in figure 6C. The difference in the levels of EZH2 recruited to ΔNp73α promoter in the different conditions is significant (pLXSN vesus LMP-1 p value = 0.038, EBVΔLMP-1 versus EBV p value = 0.008). (**E**) Primary and LCLs non-transfected or transfected with scramble (Scr) or JNK-1 siRNA were fixed and processed for quantitative ChIP analysis using an antibody against the acetylated form of histone H4 following the procedure described in the legend of Figure 6A. The data are the mean of three independent experiments. The difference in the levels of H4Ac binding to ΔNp73α promoter in the different conditions is significant (primary B cells versus LCL p value = 0.002, scramble siRNA versus JNK-1 siRNA p value = 0.04). (**F**) LCLs were cultured in absence (DMSO) or presence of 20 µM of JNK-1 inhibitor for 5 hours, fixed and processed for quantitative ChIP analysis using an antibody against the acetylated form of histone H4 or p73 following the procedure described in the legend of Figure 6A. The data are the mean of three independent experiments. The difference in the percentage of H4Ac or p73 binding to ΔNp73α promoter in the different conditions is significant (DMSO versus JNK-1 inhibitor p value = 0.004 for H4Ac and p value = 0.001 for p73). (**G**) Primary B cells and EBV-positive B cells were analyzed by immunoblotting for the levels of the indicated proteins. (**H**) Forty µg of total extract from RPMI- EBVΔLMP-1 pLXSN and pLXSN-LMP-1 cells were analyzed by immunoblotting for the levels of the indicated proteins. (**I**) Nuclear and cytoplasmic fractions from RPMI- EBVΔLMP-1 pLXSN and pLXSN-LMP-1 were analyzed by immunoblotting for the levels of LMP-1, EZH2, β-actin and PARP. (**J**) Cellular localization of EZH2 was determined by immunofluorescent in Primary B cells and LCLs. Fluorescent signal was visualized using Axioplan2 microscope from Zeiss laser microscopy. The pictures shown are representative of three independent staining.

Emerging lines of evidence show that epigenetic changes play an important role in carcinogenesis [41]. Accordingly, several oncogenic viruses, including EBV, are able to hijack the epigenetic machinery in order to de-regulate cellular gene expression, to persist in the host cell and complete the viral cycle [42]. EZH2 is a component of the Polycomb 2 complex and is able to methylate the Histone H3 on Lysine 27, leading to chromatin condensation and gene silencing [43]. Interestingly, it has been recently shown that, upon interferon alpha treatment, EZH2 inhibits ΔNp73α expression in hepato-cellular carcinoma cells (HCC) by direct binding to the p2 promoter [44]. Therefore, we next determined whether in primary B cells and LCL the observed alterations in ΔNp73α expression may be ascribed to changes in the EZH2 recruitment to p2 promoter. ChIP experiments showed that EZH2 binds the p53 RE within the p2 promoter only in primary B cells, but not in LCL (Figure 6A). A similar pattern was observed in ChIP experiments performed with an antibody that specifically recognized H3K27 methylated form (Figure 6A). In contrast, acetylation on lysine 9 of the Histone H3, a marker of transcriptionally active chromatin, was increased at the p53/p73 RE in LCLs in comparison to primary B cells (Figure 6A). In addition, p73 was more efficiently recruited to p53/p73 RE in LCLs than primary B Cells (Figure 6B). Loss of EZH2 at ΔNp73α promoter appeared to be dependent on LMP-1 expression in LCLs and RPMI cells (Figure 6C and D).

According to these results, histone H4 hyperacetylation, another event associated with active transcription, is strongly enhanced at ΔNp73α promoter in LCLs in comparison to primary human B cells (Figure 6E). JNK can induce H4 hyperacetylation to regulate gene expression [45], accordingly inhibition of JNK-1 by siRNA or chemical inhibitor in LCLs resulted in the decrease of histone H4 hyperacetylation at ΔNp73α promoter (Figure 6E and F). In addition, the recruitment of p73 to the ΔNp73α promoter in LCL is strongly reduced upon JNK-1 inhibition (Figure 6F).

Immunoblotting showed that EZH2 is weakly detected in primary B cells, while its protein levels are considerably elevated in LCLs (Figure 6G and data not shown). Similarly, expression of LMP-1 in RPMI cells infected with EBV-ΔLMP-1 led to a substantial increase in EZH2 protein levels (Figure 6H). To determine whether the increase of EZH2 levels and decrease of its recruitment to ΔNp73 promoter in LMP-1 cells was due to changes in its localization, we performed cellular fractionation experiments followed by immunoblotting. LMP-1 expression did not alter EZH2 cellular localization, which appeared to be exclusively nuclear in primary B cells and LCLs (Figure 6I). However, immuno-fluorescence experiments with an anti-EZH2 antibody highlighted a different pattern of staining in primary B cells and LCLs, indicating that LMP-1 induced a redistribution of EZH2 in the nucleus (Figure 6J). Indeed, EZH2 staining appears to be punctuated in primary B cells, while it is more diffuse in LCLs (Figure 6J).

Together the data show that ΔNp73α expression mediated by EBV LMP-1 correlates with the release of EZH2 from the ΔNp73 promoter, which results in the opening of the chromatin and in an increased access to transcription factors.

### ΔNp73α expression is required for EBV infected cells survival

**Figure 7:**
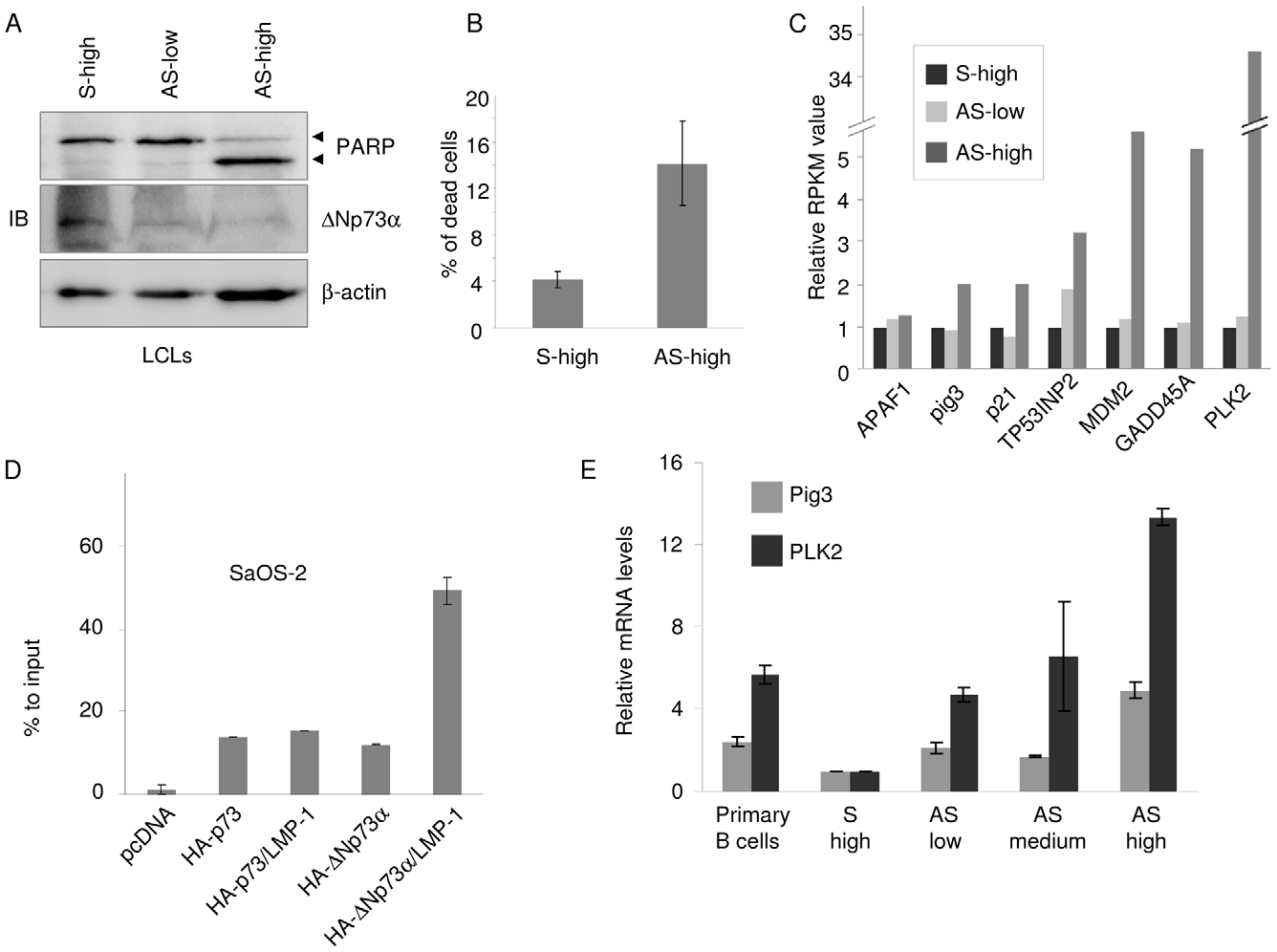
ΔNp73α inhibit expression of pro-apoptotic genes in EBV-infected cells. (**A**) LCLs were transfected with 2 µg S (S-high) and 2 increasing concentration of AS (0.5 µg, AS-low and 2 µg, AS-high), against ΔNp73α. Thirty hours after transfection cells were collected, the total lysates were extracted and analysed by immunoblotting for the indicated proteins. (**B**) Cells were treated as described in the legend of Figure 7A and live stained with PI. The percentage of PI stained cells (dead cells) were calculated by flow cytometer as explained in Material and Methods. (**C**) LCLs treated with S-high (2 µg), AS-low (0.5 µg) and AS-high (2 µg) were used to perform RNAseq. The p53 target genes which were significantly deregulated (p value<0,01 EdgeR software) in S vs. AS were represented in the histogram and expressed as relative RPMK values. (**D**) SaOS-2 cells were transfected with different pcDNA3 constructs in the indicated combinations. After 36 hours, ChIP was performed using an anti HA-tag antibody and followed by real-time PCR, using primers flanking the p53-RE BS1 within the PLK2 promoter. The percentage of binding of p73 and ΔNp73 to PLK2 promoter was determined as described in the legend of Figure 4A. (**E**) LCLs were transfected with 2 µg of ΔNp73α S (S-high) and 3 increasing concentration of ΔNp73α AS (0.5 µg, AS-low; 1 µg, AS-medium; 2 µg, AS-high). Thirty-six hours after transfection, cells were collected and processed for RNA extraction. Pig3 and PLK2 mRNA levels were determined by quantitative RT-PCR. The data are the mean of two independent experiments. The difference of Pig3 or PLK2 mRNA levels in LCLs transfected with S and AS is statistically significant (p values = 0.02 and 0.01 for Pig3 and PLK2 respectively).

To investigate the role of ΔNp73α in EBV infected cells, we down-regulated its protein levels by expressing an anti ΔNp73α anti sense oligo-nucleotide (AS). The sense oligo-nucleotide (S) was used as negative control. Figure 7A shows that ΔNp73α was efficiently down-regulated in a dose-dependent manner by AS in LCLs. Most importantly, ΔNp73α down-regulation led to PARP cleavage, indicating that ΔNp73α is involved in the inhibition of apoptosis in EBV infected cells (Figure 7A). FACS analysis confirmed that transfection with AS promoted cellular death (Figure 7B). To gain more insight into the biological significance of ΔNp73α expression in EBV infected cells, we compared the transcriptome profiling by RNA-seq of LCLs transfected by S or AS at two different concentrations. The expression levels of approximately 253 genes were found differentially expressed (214 up-regulated, and 39 down-regulated, p value<0.01) in cells transfected with S and AS (Tables S2 and S3, supplementary material). Functional analysis of the data was conducted as explained in Materials and Methods. It has been previously shown that ΔNp73α, but not ΔNp63α, plays a role in nervous system development and in the prevention of nerve growth factor-induced p53-mediated apoptosis [46]. Accordingly, we observed that the decrease in ΔNp73α levels by AS in LCL also led to the deregulation of several genes encoding products that are involved in development, e.g. in neurogenesis and in the regulation of apoptosis in neurons (Tables S2 and S3, supplementary material), indicating the specificity of our approach and validating our RNA seq analysis. Most importantly, AS ΔNp73α up-regulated genes encoding proteins, which play crucial roles in cellular transformation processes, such as apoptosis, cell cycle, DNA-repair and signaling pathways, i.e. NF-κB, Notch, RAS, and toll-like receptors (TLRs) (Table S2 and S3, supplementary material). In agreement with the functions of ΔNp73α as an antagonist of p53, several p53-regulated pro-apoptotic genes were found up-regulated in LCLs transfected by AS (Figure 7C and Tables S2 and S3, supplementary material). Interestingly, the inhibition of ΔNp73α by AS in LCLs resulted in a strong increase of *PLK2* mRNA levels. *PLK2* is a p53-regulated gene and encodes a serine threonine kinase which is involved in cell cycle regulation and cellular response to stresses. Its expression has been often found silenced by promoter methylation in Burkitt's Lymphomas [47]. ChIP experiments in SaOS-2 cells expressing HA-tagged-ΔNp73α demonstrated its binding to the promoter of *PLK2* to the p53 binding site 1, which was further increased in the presence of LMP-1, while p73 recruitment to the same promoter was not influenced by the viral oncoprotein (Figure 7D). To corroborate the RNA-seq data, we have down-regulated ΔNp73α and determined PLK2 mRNA levels by quantitative PCR. Figure 7E shows that *PLK2* and *Pig3* are down-regulated in LCL in comparison to primary B cells. In addition down-regulation of ΔNp73α resulted in a rescue of their expression confirming the RNA seq data. Another gene that was found up-regulated in ΔNp73α AS-transfected LCLs is *KLHDC8B* which also appears to be associated with lymphomagenesis and is often found mutated in familiar and sporadic Hodgkin lymphomas [48]. KLHDC8B mRNA levels were increased by 3.66 and 18.44 folds in LCLs transfected with low and high doses of ΔNp73α AS, respectively (p value = 0.001693). Taken together, these data show that in LCL, ΔNp73α plays a key role in regulating cellular genes, the products of which exert important functions in cellular transformation, including two genes, *PLK2* and *KLHDC8B*, that have been previously reported to be associated with lymphomagenesis.

## Discussion

Oncogenic viruses share the ability to target key pathways involved in preventing cellular transformation, considerably increasing the probability of an infected cell to evolve towards malignancy. One of the best characterized mechanisms of oncogenic viruses is the ability to inhibit the function of the tumor suppressor p53, a transcription factor that can trigger cell cycle arrest or apoptosis in response to stress or DNA damage [49]. Many oncogenic viruses, such as HR mucosal HPV types [15], EBV [10], [12], [14], Human T-cell Lymphotropic Virus (HTLV-1) [50], [51], Kaposi's sarcoma-associated herpesvirus (KSHV) [52]–[54] have developed strategies to inactivate p53. We have recently described a novel mechanism of deregulation of p53 transcriptional functions by the beta cutaneous HPV38 which appears, together with other beta HPV types, to be linked to skin carcinogenesis. HPV38 E7 oncoprotein promotes accumulation of ΔNp73α increasing its transcription and protein half-life ([20], [21] and our unpublished data). In turn, ΔNp73α competes with p53 for binding to p53 RE elements, preventing the activation of p53-regulated genes. Although the involvement of HPV38 in NMSC is still under debate, the demonstration that also other well-established oncogenic viruses promote ΔNp73α accumulation will further highlight the importance of the event and corroborate the potential role of HPV38 in human carcinogenesis. In this study, we show that the oncoprotein LMP-1 from EBV activates the transcription of ΔNp73, favoring the recruitment of p73 to *cis* p53 element of ΔNp73 p2 promoter. We also demonstrated that LMP-1-mediated up-regulation of ΔNp73α transcription is dependent on JNK-1, a kinase strongly activated by LMP-1. JNK-1 inhibition by different means strongly decreased ΔNp73α expression in EBV-infected cells. In addition, expression of ectopic levels of JNK-1 in the EBV-negative B-lymphoma cell line, BJAB, resulted in the activation of ΔNp73α transcription. Accordingly, LMP-1 mutants lacking the JNK-1 activating domain (CTAR2) did not influence the ΔNp73α expression levels. It is well established that several amino acid residues of p73 are phosphorylated by JNK-1 [34]. Therefore, it is likely that p73 recruitment to the ΔNp73 promoter is mediated by its JNK-1-dependent phosphorylation. Additional experiments are required to confirm this hypothesis and establish whether the p73 affinity for ΔNp73 promoter is determined by phosphorylation of one or more specific amino acids. Previous studies have shown that inhibition of JNK-1 in LMP-1 expressing cells led to decrease of cdc2 levels and cell cycle arrest [55]. In our experimental model the inhibition of cdc2 in LCLs by the chemical inhibitor roscovitine slightly affected ΔNp73α levels (data not shown).

JNK-1 could also induce ΔNp73 transcription by an alternative mechanism via activation of the proto-oncogene c-Jun. It has been shown that p73 acts in synergistic manner with c-Jun in promoting cellular survival [56]. This event is well explained by their cooperative ability to activate the transcription of specific subsets of cellular genes. ChIP-seq experiments have revealed the presence of AP1-binding motifs in close proximity to the p73 *cis* elements in promoters of genes encoding proteins with anti-apoptotic functions [57]. Brigati *et al*. have shown that TPA treatment of Germinal Center B cells, able to induce ΔNp73α expression, also leads to binding of c-Jun to an AP1 site which was located on the promoter of ΔNp73α, just upstream the p53/p73 RE [28]. According to these findings, we observed that in LCLs c-Jun is recruited to an AP1 *cis* element closely located to the p53/p73RE of ΔNp73 promoter (our unpublished data).

ChIP experiments in primary and EBV-immortalized B cells showed that activation of ΔNp73 promoter by the recruitment of p73 correlated with the displacement of the polycomb 2 complex component EZH2 and epigenetic changes. The apparently paradoxical finding that EBV infected B cells, despite the increased intracellular levels of EZH2, show reduced amount of EZH2 and lower levels in H3K27 methylation on the promoter of ΔNp73, recalls the scenario observed in HPV16 E6/E7 expressing cells [58]. Hyland *et al*. observed increased levels of EZH2 in the presence of HPV16 E6 and E7 proteins, which correlated with a decrease of H3K27 methylation. The authors explained that this phenomenon was due to an increase in KDM6A and KDM6B levels, two demethylase enzymes, and a decrease in BMI1, a Polycomb1 protein which stabilizes Polycomb 2-mediated methylation. According to this model, EBV is able to trigger accumulation of KDM6B via LMP-1 ([59] and our unpublished data) as well as a reduction of BMI1 levels (our unpublished data). Accumulation of EZH2 in cells expressing LMP-1 could be a consequence of post-translational modifications that negatively regulate its enzymatic activity. Accordingly, it has been previously shown that phosphorylation of EZH2 by AKT on serine 21 suppresses methylation of lysine 27 in Histone 3 [60]. It has been reported that EBV LMP-1 triggers the AKT pathway [61] which is often found activated in NPC and Hodgkin's lymphomas [62], [63]. Based on these findings, we could speculate that the loss of EZH2 recruitment to ΔNp73 promoter is due to serine 21 phosphorylation. We are currently assessing this hypothesis.

High levels of p73 and ΔNp73 have been observed in B cell chronic lymphocytic Leukemia [64]. Although resting B cells do not express ΔNp73, epigenetic changes leading to ΔNp73 up-regulation were observed in the activated B cells compartment of the germinative center of the tonsil. Thus, it is likely that the mechanisms characterized in EBV-infected cells in this study may also occur in different scenarios independently of the presence of the viral oncoprotein.

To evaluate the biological significance of EBV-mediated ΔNp73α over-expression in the transformation of B cell, we down-regulated ΔNp73α expression by AS in LCL, and compared the cellular expression profiling with one of the S transfected LCL. Decrease in ΔNp73α levels led to the alteration of the expression of cellular genes linked to neurogenesis as well as to the regulation of apoptosis in neurons. These results are consistent with the known *in vivo* functions of ΔNp73 in brain development and in the prevention of nerve growth factor induced p53-mediated apoptosis [46]. An additional cluster of genes that appeared down-regulated by ΔNp73α in LCL is a group of Homeobox genes. To our knowledge, this is the first time that ΔNp73α has been shown to be implicated in the regulation of HOX genes. Since both HOX genes and ΔNp73α are aberrantly expressed in cancer cells, these findings, if confirmed, could further contribute to the understanding of the events associated with carcinogenesis [22], [65].

Loss of *PLK2* expression by promoter CpG island methylation is one of the most common epigenetic events in B-cell lymphomas [47]. It is worth noting that we found *PLK2* gene strongly up-regulated upon inhibition of ΔNp73α in LCL. It is well known that *PLK2* promoter is positively regulated by p53. Thus, it is highly likely that ΔNp73α induces *PLK2* down-regulation by altering the p53 transcriptional function. For the first time, our data provide evidence for a link between *PLK2* expression silencing and ΔNp73α in EBV-infected cells. Our findings also suggest that LMP-1 increases the affinity of ΔNp73α for PLK2 promoter without altering its protein levels (HA-ΔNp73α: Figure 7D and our unpublished data). It is possible that the viral oncoprotein, independently of its ability to positively regulate the ΔNp73α transcription, may increase ΔNp73α affinity for PLK2 promoter by promoting post-translational modifications.

Similarly to *PLK2*, *KLHDC8B* has been linked to lymphomagenesis. Multiple cases of Hodgkin's lymphoma with translocations or polymorphisms affecting *KLHDC8B* have been reported in the same family [66], indicating that inhibition of its expression plays a role in B cell transformation. Our data show that the expression of *KLHDC8B* was restored upon ΔNp73α down-regulation. According to the well characterized ΔNp73α ability to act as an inhibitor of p53, the expression levels of different p53 responsive genes (*MDM2, APAF1, GADD45, BAX, CCND1,* etc.) increased significantly after ΔNp73 down-regulation, leading to apoptosis. Other subgroups of genes that resulted to be regulated by ΔNp73α are the ones involved in lymphocyte migration and proliferation, cytokine production and innate immune response. As a whole, the RNA seq data indicate that in EBV infected cells ΔNp73α may contribute to the development of EBV-associated disease in several ways, e.g. by inhibiting apoptosis, promoting B cell growth, as well as by modulating host defence machinery allowing EBV persistence.

In summary, our data underline the important function of ΔNp73α in EBV-induced cellular transformation, unveiling novel links between its accumulation and deregulation of the expression of many cellular genes. However, the degree to which ΔNp73α oncogenenic effects exceed tumor suppressor effects of p73 activation remains to be better determined.

## Materials and Methods

### Expression vectors

Cellular and viral genes were expressed using the retroviral vector pLXSN (Clontech, Palo Alto, CA) or the expression vector pcDNA-3 (Invitrogen). The pLXSN-LMP-1 construct has been previously described (68). The constructs pLXSN-LMP-1 mutants (LMP-1AxAxA, LMP-1 378 STOP, and LMP-1AxAxA/378STOP) were generated in this study using standard molecular biology techniques. The constructs pcDNA3 HA-ΔNp73α, pcDNA3 HA-p73α were previously described [20].

### Cell culture procedures

RPMI 8226 cells (harbouring mutated p53: Glu 285 to Lys) (RPMI) were kindly provided by Dr Christophe Caux (Centre Léon Bérard, Lyon, France). RPMI pLXSN or pLXSN-LMP-1 were generated as described in Fathallah *et al.* [67]. RPMI EBV and EBVΔLMP-1 were obtained as in [68]. Expression of LMP-1 wild-type or LMP-1 378 stop mutant in RPMI EBVΔLMP-1 was achieved by transduction with recombinant retroviruses [67]. The EBV-immortalized lymphoblastoid cell lines (LCLs), ATM14, ATM11, 48513F, 48513M, 2095, 2145 and EBV negative immortalized B cells, BJAB (with mutated p53: His 193 to Arg), were previously described [69] (or generated at IARC, Lyon France). Several LCLs were generated in this study by infecting primary B cells isolated from different donors as previously described [67]. Primary and immortalized B cells were cultured in RPMI 1640 medium (GIBCO; Invitrogen life Technologies, Cergy-Pontoise, France) supplemented with 10% FBS, 100 U/ml penicillin G, 100 mg/ml streptomycin, 2 mM L-glutamine, and 1 mM sodium pyruvate (PAA, Pasching, Austria). SaOS-2 and human embryonic kidney cells (HEK293) were cultured in foetal calf serum (FCS) and Dulbecco's modified Eagle medium (DMEM) (Gibco) using standard culturing conditions. Cells were treated with JNK inhibitor SP600125 at the final concentration of 20 µM. DNA plasmids were transiently transfected in cells by using FuGENE6 or Xtreme gene 9 reagents (Roche) according to the purchased protocol.

Immuno-fluorescence in primary and immortalized B cells was performed as described in Fathallah *et al*. [67].

For FACS staining, cells were collected and washed twice in PBS, then stained with Propidium iodide (PI) at the final concentration of 5 µg/ml. Subsequently, cells were analyzed for the % of dead cells by FACS CANTO (Becton Dickinson).

### Gene expression silencing

Gene silencing of SAPK/JNK was performed using SignalSilence SAPK/JNK siRNAI (6269 cell signalling). Cells (8×10^5^) were transfected with siRNA to the final concentration of 100 nM by oligofectamine (invitrogene) according to the manufacture protocol. p53 gene silencing was performed as in Accardi *et al.* [20]. ΔNp73α levels were down-regulated by electroporating 1.5×10^6^ cells with either 0.5 or 2 µg of AS or S oligos (for AS and S oligos sequences please see [20]). Cells electroporation was performed by Neon Transfection System, using a pulse voltage of 1350 v and a pulse width of 30 ms. To specifically silence p73 isoform we used the target sequence: 5′-CAGACAGCACCTACTTCGA-3′ spanning from +71 to +90 bp downstream of the transcription start codon was cloned in OmicsLink shRNA Expression system containing a puromycin selection marker (HSH018180-6-HIVmH1, OS395979, GeneCopoeia). As negative control, a scrambled shRNA (sH1) was used. Lentivirus production was performed as previously described [70]. Lentiviral suspension was added to 1.5×10^6^ LCLs and selection with puromycin (0.8 µg/ml) was initiated 24 hours later. One week post infection cells were collected and processed for the different experiments.

### RT-PCR and Quantitative PCR

Total cellular RNA was extracted from cells using the Absolutely RNA Miniprep kit (Stratagene). RNA Reverse transcription to cDNA was carried out by RevertAid H Minus M-MuLV Reverse Transcriptase (MBI Fermentas) according to manufacturer's protocol. Quantitative PCR (Q-PCR) was performed in duplicate in each experiment as previously described [67]. The primer sequences used for RT and Q-PCR are indicated in Table S1A (supplementary material).

### Immunoblotting and antibodies

Preparation of whole or fractioned cell lysates extracts, sodium dodecyl sulfate -polyacrylamide gel electrophoresis (PAGE), and immunoblotting (IB) were performed as previously described [20], [21]. The following antibodies were used for IB: β-actin (C4; MP Biomedicals), human p53 (NCL-CM1; Novocastra Laboratories Ltd.), p73 (anti-p73 Ab-1; Calbiochem), hemagglutinin (HA)-peroxidase-high affinity (3F10; Roche), anti-LMP-1 Ab (S12; a gift from Georges Mosialos, Alexander Fleming Institute, Varkiza, Greece), PARP (9542; Cell signalling), SAP/JNK1(56G8; Cell signalling), anti-EZH2 (AC22; Cell signalling).

### DNA pull-down

A biotinylated fragment of ΔNp73 promoter was generated by PCR using the genomic DNA as template and a biotinylated primer Fw 5′-Btndt CTGGTGGGTTTAATTA-3′ and a non-biotinylated primer Rev 5′- AGGAGCCGAGGATGCTGG-3′ (Sigma). Cells were re-suspended in lysis buffer HKMG (10 mM HEPES, pH7.9, 100 mM KCl, 5 mM MgCl2, 10% Glycerol, 1 mM DTT and 0.5% NP-40) incubated in ice for 10 min, then lysed by sonication (25% Amp, 1 min). One mg of total cellular extracts was pre-cleared with 40 µl of streptavidine-agarose beads (Amersham Bioscience) for 1 hour at 4°C, and then incubated with 2 µg of purified DNA biotinylated probe for 16 hours at 4°C. Poly dI-dC (40 µg) was added to the reaction to avoid unspecific binding. DNA bound proteins were recovered by incubating with 60 µl of streptavidine-agarose beads for 1 hour at 4°C and washed several times with HKMG buffer [71]. Beads were resuspended in 1× SDS-PAGE loading buffer and analyzed by immunoblotting.

### Chromatin immunoprecipitation

Chromatin immunoprecipitation (ChIP) was performed with Diagenode Shearing ChIP and OneDay ChIP kits or with LowCell ChIP Kit according to the manufacturers' protocols, using the following antibodies: EZH2 (AC22; Cell signalling), Histone 3 Lysine 27 Trimethyl polyclonal antibody H3K27 (Epigentek), Histon 3 acetylated lysine 9 antibody H3K9Ac (9649S; cell signalling), Acetyl-Histone H4 (17–630; Millipore), HA hemagglutinin (HA) high affinity (3F10; Roche), and p73 (anti-p73 Ab-1; Calbiochem). The eluted DNA was used as template for Quantitative PCR as previously described [21]. Primers for Quantitative ChIP are listed in Table S1B (supplementary material).

### RNA sequencing

RNA integrity and quantification of the total cellular RNA from LCLs tranfected with ΔNp73α AS and S oligo-nucleotide were characterized by measuring the 28s/18s rRNA ratio and RIN using the Agilent 2100 bioanalyzer instrument, and the Agilent RNA 6000 Nano kit. 5 µg of total cellular RNAs was depleted from rRNA using the Invitrogen RiboMinus Eukaryote kit according to manufacturer's standard protocol. The absence of 28s/18s rRNA was checked on the Agilent 2100 bioanalyzer instrument. Five hundred ng of each sample were enzymatically fragmented using 1 unit of RNase III provided in the SOLiD Total RNA-seq Kit, incubated at 37°C for 10 minutes and cleaned up using the Invitrogen RiboMinus Concentration Module according to manufacturer's standard protocol. RNA yield and size distribution were assessed with the Agilent 2100 Bioanalyzer instrument and the Agilent RNA 6000 Pico kit. The amplified whole transcriptome library for each sample was constructed according to Lifetechnologies's SOLiD Total RNA-seq Kit protocol (PN 4452437 Rev.B). To summarize, adaptors were hybridised and ligated to 100 ng of fragmented rRNA-depleted total RNAs followed by the construction and subsequent purifications of cDNAs using successively the SOLiD Total RNA-seq Kit and the Agencourt AMPure beads. cDNAs were then barcoded, amplified with 15 cycles of PCR and purified using the Invitrogen PureLink PCR Micro Kit. Yield and size distribution of the amplified DNA libraries were assessed with the Agilent 2100 Bioanalyzer instrument and the Agilent DNA 1000 kit. After minimizing the DNA in the 25–200 bp range, 0.4 pM of each barcoded libraries were pooled at equimolar concentrations prior to template bead preparation, in which the pooled library is clonally amplified by emulsion PCR following the Lifetechnologies's SOLiD EZ bead E80 protocols (PN 4441486 Rev. D, 4443494 Rev. D, 4443496 Rev. D). Two hundred and forty µl of emPCR beads were 3′ modified and deposited on 4 lanes FlowChip before being incubated for 60 minutes at 37°C. The forward 50 bp reads sequencing chemistry was applied.

### RNA seq data analysis

The secondary and tertiary analyses was done with LifeScope software v. 2.5.1 from Life Technologies (Build ID:LifeScope-v2.5.1-r0_102906_20120406100430)

The raw data (xsq files) from each lane were grouped per sample (based on the barcodes) before launching the standard RNA seq workflow on the 3 samples (EBV_sense, EBV_antisens1, EBV_antisens2). We kept all the standard parameters as advised by Life Technologies.

This workflow includes 3 modules: the “mapping analysis” for which we used hg19 as reference genome, the “coverage analysis” and the “count known genes and exons analysis”.

After reads mapping, the R/Bioconductor package edgeR (empirical analysis of digital gene expression data in R) was used to study differential gene expression [72]. After fitting a negative binomial model, data obtained from antisense samples were grouped before applying the “common dispersion” function in edgeR. Next, differential gene expression was determined using the exact test. Heatmaps and gene set expression comparisons were performed with BRB-ArrayTools software Version 4.2.1. To this end, reads were RPKM (Reads Per Kilobase of exon model per Million mapped reads) normalized and corresponding gene lists were filtered for selected pathways (Table S3, supplementary material).

### Statistical analyses

Statistical significance was determined by Student T test. The p value of each experiment is indicated in the corresponding Figure legend. Error bars in the graphs represent the standard deviation.

## Supporting Information

Table S1 Primer sequences used for PCR (A) or ChIP (B) experiments.
(DOC)

Table S2 List of genes that are altered in their expression upon down regulation of ΔNp73α in LCL.
(XLSX)

Table S3 Differently regulated Genes (RPMK normalized read counts significantly changing in S vs. AS with a p value<0.01, EdgeR) were submitted to BRB (BRB-ArrayTools, NCI) and David software (DAVID Bioinformatics Resources 6.7, NIH) to search for functional annotation clusters and pathways.
(DOC)
