# Supplementary material for: Epstein - Barr Virus Transforming Protein LMP-1 Alters B Cells Gene Expression by Promoting Accumulation of the Oncoprotein ΔNp73α
Source: PLoS Pathog. 2013 Mar 14;9(3):e1003186. doi: 10.1371/journal.ppat.1003186 (PMC3597522; doi:10.1371/journal.ppat.1003186)
Supplement: Table S1 — Primer sequences used for PCR (A) or ChIP (B) experiments. (DOC) [file ppat.1003186.s001.doc]

| Gene | Forward | Reverse |
| --- | --- | --- |
| GAPDH | 5’-AAGGTGGTGAAGCAGGCGT-3’ | 5’-GAGGAGTGGGTGTCGCTGTT-3’ |
| LMP1 | 5’-CCCCCTCTCCTCTTCCATAG-3’ | 5’-GCCAAAGATGAACAGCACAA-3’ |
| EBER2 | 5’-CCCTAGTGGTTTCGGACACA-3’ | 5’-ACTTGCAAATGCTCTAGGCG-3’ |
| ΔNp73 | 5’-AACCATGCTGTACGTCGGTGACCCC-3’ | 5’-GCGACATGGTGTCGAAGGTGG -3’ |
| p73 | 5’-ATGGCCCAGTCCACCGCCAC-3’ | 5’-GCTGCTCAGCAGATTGAACT-3’ |
| p53 | 5’-CCCAAGCAATGGATGATTTGA -3’ | 5’-GGCATTCTGGGAGCTTCATCT-3’ |
| JNK-1 | 5’-TTGGAACACCATGTCCTGAA-3’ | 5’-ATGTACGGGTGTTGGAGAGC-3’ |
| ΔNp73 α | 5’-ATGCTGTACGTCGGTGACCCC -3’ | 5’-TAACAGGATTGGGGTGTCCAAACTG -3’ |
| ΔNp73 β | 5’-ATGCTGTACGTCGGTGACCCC -3’ | 5’-CTCGTCAGGACCTGGGGGCCCT -3’ |

Table 1A.

Table 1B.

| Promoter | Forward | Reverse |
| --- | --- | --- |
| ΔNp73 (p53 RE) | 5’-CTGGTGGGTTTAATTATGGAGCC-3’ | 5’-AGGAGCCGAGGATGCTGG-3’ |
| ΔNp73 (AP1 RE) | 5’-CCAAGACGGCTGAAATACAATGG-3’ | 5’-AAAGCAGGGGTGAGTCCAAGAC-3’ |
| PLK2 (BS1) | 5’-CTTAGGCCACAAATGAATACA-3’ | 5’-TCCCAGATTTGTGCAAACCT-3’ |
