# Supplementary material for: Epstein - Barr Virus Transforming Protein LMP-1 Alters B Cells Gene Expression by Promoting Accumulation of the Oncoprotein ΔNp73α
Source: PLoS Pathog. 2013 Mar 14;9(3):e1003186. doi: 10.1371/journal.ppat.1003186 (PMC3597522; doi:10.1371/journal.ppat.1003186)
Supplement: Table S3 — Differently regulated Genes (RPMK normalized read counts significantly changing in S vs. AS with a p value<0.01, EdgeR) were submitted to BRB (BRB-ArrayTools, NCI) and David software (DAVID Bioinformatics Resources 6.7, NIH) to search for functional annotation clusters and pathways. (DOC) [file ppat.1003186.s003.doc]

| Cellular events | Genes |
| --- | --- |
| Apoptosis | *TP53BP2, STK17A, MDM4, BCL2, MYD88, CASP10, BCL2A1, BNIP3, CASP1, DAP3, DAXX, CASP4, PSEN2, DFFA, STK17B, BAX, PAWR, BCL2L1, CASP8DAP, BCL10, TNFAIP1, CASP2, BAD, DAPK3, PDCD1, BAK1, CASP5, BCL2L2, NAIP, BNIP1, APAF1, BAG4, BNIP2, CASP6, PDCD2, MCL1, CASP9, CASP3, CASP7* |
| DNA damage | *MPG, MGMT, DDB1, INPPL1, UNG, PRKDC, RFC3, LIG3, MSH2, RAD54L, FANCG, XRCC5, TOP2A, DFFA, ERCC5, TOP2B, MSH3, NTHL1, PMS2, PRIM1, MLH1, RFC4, RAD51, BLM, RAD51L1, ATM, FANCB, RAD17, RAD21, FANCA, ERCC3, POLB, RAD51L3, BRCA1, RAD23B, RAD1, FANCC, XRCC2, PMS1, ERCC1, XRCC1, ERCC2, XRCC3, LIG1, XRCC4, XPA, BRCA2, TP53, TERT, MSH5, TDG, RAD51C, RAD23A, DNMT1, WRN, TOP3B, ERCC6, TOP1, LIG4, DDIT3, IGHMBP2, MSH4, DDB2, RAD52, XPC* |
| Chromatin organization | *CHD2, EPC1, HISTH2AA, HISTH2BA* |
| p53 signaling | *CCND1, MDM2, GADD45A, TIMP3, BCL2, CDKN1A, CDK2, CDK4, CCNE1, E2F1, TP53, BAX, PCNA, ATM, APAF1, RB* |
| Cell cycle | *DHRS2, PGF, GDNF, KLF4, SLC5A5, EFCAB10, MYO1A, PHYHIP, PLK2, KANK3, COL1A1, TNFRSF10D, MYT1, KCNN1, USP2, TMEM146, CACNA1G, SLC32A1, LAMA1, HSPA6, PPL, TRIM55, SLC7A5P1, C6orf58, UBASH3A, NR1D1, RAP1GAP, PADI4, PLXNB3, GRHL3, KITLG, SAA2, SERPINA6, EPB41L4B, PLA2G4C, UNC13A, GDF15, CYP4F3, EPHA2, OTP, PDGFRA, GHRL, VWCE, PPP1R15A, LIF, LCE1C, HMOX1, RSPH1, SIGLEC5, SIGLEC14, ZFHX2, GEM, NR1D2, JUN, CELSR3, SCNN1G, EPHB2, LCE1E, GPR56, EDN2, TNFRSF10C, CYP1A1, FOS, PLIN4, IL27, RAGE, ATF3, TPPP, MUC17, RGAG4, PVRL4, DFNB31, AFAP1, USHBP1, SNAI1, GCGR, GRIN1, NCAN, ESPN, NEURL3, DPEP2, DNAH12, ALDH1A3, RRAD, IP6K3, CCDC65, DNAH3, DUSP8, LOC643201, SYT5,DUOX1, IL6, FOXJ1, CSF3, ULBP1, HIST1H2BA, LOC386758, GPR87, MAFB, ALOX12B, LPAL2, RASD1, CDKN2B, LRRC48, CYP4F2, CXCL3, PGLYRP2, C1orf68, LRRC66, COL2A1, SRL, TMEM151B, NGFR, TMEM27, CCDC149, GRAMD2, FBLN5, PCDH8, GNG4, ARHGEF33, GOLT1A, ATG9B, C8orf75, C7orf53, TRIM29, TMEM52, SP6, NPW, CALR3, NKD2, AKR1B15, SNORA69, ST6GALNAC3, C6orf138, ASTL, IDI2-AS1, BAAT, KIAA1199, C2,OAZ3, SEMA3B, SNORA2B, EEF1A2, C1S, PLXNA4, ZNF833P, KIAA1683, PKP2, TNXB_dup2, IGLL3P, STK32B, JAZF1, WHAMML1, SMOC1, IRGM, SORCS2, C14orf79, TJP1, KLHDC8B, AGAP11, C14orf182m, SHE, FOSB, SERPINE1, C6orf27, CYP2E1, SCNN1B, PLA2G4E, ATP4A, BAI2, TTTY15, UTY, KDM5D, RPS4Y1, USP9Y, MIR1229, CTXN1, SNORA27, RNU6ATAC, C3orf47, NLGN2, IL13, C21orf122, STON1-GTF2A1L, SNORA53, GPSM1, LRRC14B, FAM66D, SNORA42, TLR5, SCARNA11, SNORA41, SNORA74A, RNU12, SNORA65, SNORA64, SNORA74B, SNORA23, SNORA36A, SCARNA4, SNORA13,DDX3Y, EIF1AY* |
| NF-κB signaling | *TNFRSF1B, MYD88, NFKBIA, TNF, MAP3K1, IKBKB, TNFRSF1A, RIPK1, TAB1, FADD, TRADD, CHUK, IKBKG, MAP3K7, NFKB1, TRAF6, RELA, MAP3K14, IL1R1, IL1A, TNFAIP3* |
| Innate immuneresponse | *IRAK2, TICAM1, TLR3, TLR5, TLR7, IRGM, IL27, PGLYRP2, IL1RAPL2, DMBT1, FOS, JUN, EIF2AK2, MAP2K6, MYD88, NFKB1, TRAF6, RELA, MAP3K14, TAB1, ELK1, CHUK, IKBKG, MAP3K7, MAPK14, PPARA, MAPK8, MAP2K3, NFKBIA, IKBKB, MAP2K4, MAP3K1* |
| Jak/STAT pathway | *IL10RA, IL6ST, LIF, Pim1* |
| Homeobox genes | *SATB1, DLX2, EMX1, ZEB2, ZFHX2, OTP*, *Tgif1, HOXB9, HOXC13, PKNOX2, EN2* |
| Nervousgenesis, symaptic transmission, neuron apoptosis | *ALDH1A3, IL6, CDKN1C, CELSR3, DLX2, DFNB31, DSCAML1, DRD2, ESPN, EPHB2, EMX1, FOXG1, GDNF, GNAT2, GHRL, NCAN, KCNN1, SCNN1B, OTP, SCNN1G, SLC32A1, SLC6A9, SLC30A3, TAS2R30, FOS, LIF, KAL1, JUN, NGFR, NR4A2, SEMA3B, ZEB2* |
| Lymphocytes migration, proliferation and cytokines synthesis | *CSF3, IL6, IL22RA1, IL9R, CXCL3, CXCL2, KITLG, IL13, LIF, TNFRSF10C, TNFRSF10D, PDGFRA, NGFR, IL27 FOXJ1, TAC* |
